# Registration of published randomized trials: a systematic review and meta-analysis

**DOI:** 10.1186/s12916-018-1168-6

**Published:** 2018-10-16

**Authors:** Ludovic Trinquart, Adam G. Dunn, Florence T. Bourgeois

**Affiliations:** 1 Department of Biostatistics, Boston University School of Public Health, Boston, Massachusetts USA; 2 Centre for Health Informatics, Australian Institute of Health Innovation, Macquarie University, Sydney, Australia; 3 Department of Pediatrics, Harvard Medical School, Boston, Massachusetts USA; 4 Center for Pediatric Therapeutics and Regulatory Science, and Computational Health Informatics Program, Boston Children’s Hospital, Boston, MA USA

**Keywords:** Randomized controlled trials, Registration, Reporting bias

## Abstract

**Background:**

Prospective trial registration is a powerful tool to prevent reporting bias. We aimed to determine the extent to which published randomized controlled trials (RCTs) were registered and registered prospectively.

**Methods:**

We searched MEDLINE and EMBASE from January 2005 to October 2017; we also screened all articles cited by or citing included and excluded studies, and the reference lists of related reviews. We included studies that examined published RCTs and evaluated their registration status, regardless of medical specialty or language. We excluded studies that assessed RCT registration status only through mention of registration in the published RCT, without searching registries or contacting the trial investigators. Two independent reviewers blinded to the other’s work performed the selection. Following PRISMA guidelines, two investigators independently extracted data, with discrepancies resolved by consensus. We calculated pooled proportions and 95% confidence intervals using random-effects models.

**Results:**

We analyzed 40 studies examining 8773 RCTs across a wide range of clinical specialties. The pooled proportion of registered RCTs was 53% (95% confidence interval 44% to 58%), with considerable between-study heterogeneity. A subset of 24 studies reported data on prospective registration across 5529 RCTs. The pooled proportion of prospectively registered RCTs was 20% (95% confidence interval 15% to 25%). Subgroup analyses showed that registration was higher for industry-supported and larger RCTs. A meta-regression analysis across 19 studies (5144 RCTs) showed that the proportion of registered trials significantly increased over time, with a mean proportion increase of 27%, from 25 to 52%, between 2005 and 2015.

**Conclusions:**

The prevalence of trial registration has increased over time, but only one in five published RCTs is prospectively registered, undermining the validity and integrity of biomedical research.

**Electronic supplementary material:**

The online version of this article (10.1186/s12916-018-1168-6) contains supplementary material, which is available to authorized users.

## Background

Prospective clinical trial registration is at the foundation of research transparency [1, 2]. By documenting the existence of clinical trials and providing a summary of protocol details before patients are enrolled and trial results become known, registries can prevent unnecessary duplication of trials, facilitate the identification of research gaps, and support coordination of study efforts for a disease [3]. As registration allows public scrutiny of the availability of trial results, it also provides means to identify and monitor biased reporting of trials. Several cases have highlighted how selective reporting can lead to considerable harm to patients. Rofecoxib, a nonsteroidal anti-inflammatory drug, was found to be associated with increased risk of myocardial infarction as early as 2000, but the primary trial publication selectively omitted findings on cardiovascular safety, and rofecoxib was not withdrawn from the market until several years later, in 2004 [4, 5]. Incomplete trial reporting can also lead to considerable waste of resources, as has been seen in the case of oseltamivir [6] or gabapentin [7, 8] with billions of dollars spent despite poor evidence of efficacy. Prospective trial registration—if universally implemented—can serve as a powerful tool to detect and prevent this type of publication bias and selective outcome reporting [3, 9–11].

The International Committee of Medical Journal Editors (ICMJE) announced in 2004 a requirement for prospective registration of clinical trials as a pre-requisite for consideration for publication in its member journals, beginning the following year [2]. In the USA, the Food and Drug Administration Amendments Act (FDAAA) has mandated prospective trial registration with ClinicalTrials.gov since 2007 for drugs, biologics, and devices subject to Food and Drug Administration (FDA) regulation. The requirements for registration and result posting have recently been expanded with the FDAAA Final Rule and a similar policy by the National Institutes of Health [12, 13]. Other countries and funders have implemented similar policies [14, 15].

The clinical trial research community has widely adopted trial registration, and trial registries have been leveraged to monitor research activity and integrity [16–18]. For example, trial registration data have been used to show that the results of registered trials are frequently not disseminated, either through reporting in biomedical journals or posting on ClinicalTrials.gov [19, 20]. However, several studies in specific medical specialties have suggested that many published trials are not registered or not registered prospectively, raising concerns that long-standing efforts have not succeeded in achieving universal trial registration [21–23]. Our objective in this systematic review was to determine the extent to which published randomized controlled trials (RCTs) were registered and registered prospectively.

## Methods

### Search and study selection

Studies were eligible if they were based on a sample of RCTs identified in published reports in medical journals and evaluated their registration status. All eligible studies across medical specialties were included. Studies not limited to RCTs were not eligible, unless they provided relevant data on registration of the subgroup of RCTs. Studies that determined trial registration only through mention of trial registration in the published article (i.e., the article included the trial registration number in the abstract or main text), but did not further search for information on registration status with either a search for trial records in registries or by contacting the trial investigators to inquire about registration status, were excluded. Lastly, studies that covered a majority of trials published prior to 2005 (i.e., the middle of the range of years covered was prior to 2005) were excluded.

We did not register a protocol for the review. We searched MEDLINE via PubMed and EMBASE without language restriction for studies published between January 1, 2005, and October 31, 2017. The search strategy was (trial[tiab] OR trials[tiab]) AND (registration[ti] OR registered[ti] OR unregistered[ti]) in MEDLINE and (trial:ti OR trial:ab OR trials:ti OR trials:ab) AND (registration:ti OR registered:ti OR unregistered:ti) in EMBASE. Additionally, we screened all articles that were cited by or that cited any of the included studies and studies excluded after full-text screening, and we screened the reference lists of related reviews [24–26] for additional eligible studies.

Two investigators screened the titles and abstracts of all records, independently and in duplicate, to identify potentially eligible studies for further assessment. Discrepancies were discussed to reach consensus. All authors then independently assessed each remaining full-text article for inclusion. We again reviewed all discrepancies, and the final list of included studies was determined by consensus among all authors.

To eliminate overlapping samples of RCTs, we compared the medical specialties, journals searched for published RCTs, and time periods covered by the studies. In cases of complete overlap (i.e., when a study sample was included in a more recent, larger study), we discarded the smaller study encompassed in the larger one. When RCTs were identified based on a specific clinical topic (e.g., RCTs of cognitive behavioral therapy and new-generation antidepressants), we considered that the study was unlikely to overlap with another study based on a search of journals of a relevant medical specialty (e.g., journals in psychology), after consideration of years and journals searched. We could not exclude the possibility that some studies that searched for RCTs across multiple specialties or in general medicine journals would not overlap with other studies. To address this, we discarded studies for which we could not completely ensure that there was no overlap from the primary analysis but included the totality of the available data in a secondary analysis.

### Data extraction

Two investigators independently extracted data from each included study using a standardized data collection form. Discrepancies were resolved by consensus. For each included study, we extracted the medical specialty, the publication years of included RCTs, the number of journals searched for RCTs, the list of journals (when provided), the number of identified RCTs, and the number of registered RCTs. We also assessed how trial registration was assessed (i.e., through the reporting of a trial registration number in the article, by searching for trial records in trial registries [27], and/or by contacting corresponding authors). We noted which registries were searched and assessed whether studies had included trials that started enrolment before 2005, as the ICMJE policy was implemented in September 2005. Moreover, we noted if each study assessed if the trial registration was prospective. In that case, we also extracted the number of trials registered prospectively. According to the ICMJE, trials must register at or before the onset of patient enrollment as a condition of consideration for publication. According to FDAAA, Applicable Clinical Trials must be registered no later than 21 days after enrollment of the first participant. We considered trials to be registered prospectively, as defined by the authors of the review.

Lastly, we also extracted the number of registered trials in certain subgroups, when this information was available: in trials that started enrolment after 2005, according to publication year and trial size, and in industry-supported trials. We defined industry support as direct or indirect financial support by a company that produces drugs or medical devices.

### Data synthesis

For each included study, we calculated the proportion of registered RCTs with the 95% Clopper-Pearson exact confidence interval. We assessed the heterogeneity across studies through a visual examination of a forest plot and heterogeneity statistics (Cochran’s chi-square test and between-study variance *τ*^2^). We estimated pooled proportions by using arcsine transformations and a beta-binomial random-effects model with Anscombe continuity correction [28, 29]. We examined potential small-study effects by using a funnel plot showing the relationship between the log odds of being registered and the associated standard error. Similar methods were used for the synthesis of prospective registration data. For the latter analysis, we first calculated the proportion of RCTs registered prospectively out of the total number of identified RCTs. We also calculated the proportion of RCTs registered prospectively among registered RCTs.

To examine the effect of time on registration prevalence, we conducted three analyses. We synthesized data for RCTs that started enrolment exclusively after 2005. Because the RCT start dates were frequently unclear, we also performed an analysis limited to RCTs published in 2010 or later in order to capture RCTs that were likely to have all been initiated after the implementation of the ICMJE registration policy. The year 2010 was chosen based on data indicating that the time from start of study enrollment to publication is approximately 5 years [30, 31]. Finally, in order to detect changes in registration over the study period, we examined the proportion of registered trials by publication year, within studies and across studies, by using meta-regression models. We fitted the models on the log odds scale and back transformed the fitted lines to produce a plot showing the proportion of registered trials against publication year. We also analyzed the proportion of prospectively registered trials by publication year through a meta-regression.

To further explore sources of heterogeneity in registration prevalence, we performed a subgroup analysis in trials (fully or partially) supported with funding from industry. We also examined the prevalence of registration according to the sample size of RCTs. Because different thresholds were used across studies, we reported the extracted data without meta-analysis. Finally, we examined the subset of studies that identified published RCTs from a sample of high-impact factor journals. Analyses were performed using R v3.4.1 (R Development Core Team, Vienna, Austria) with the metafor package for meta-analysis. The data and R code are provided in Additional files 1 and 2.

## Results

### Characteristics of included studies

**Fig. 1:**
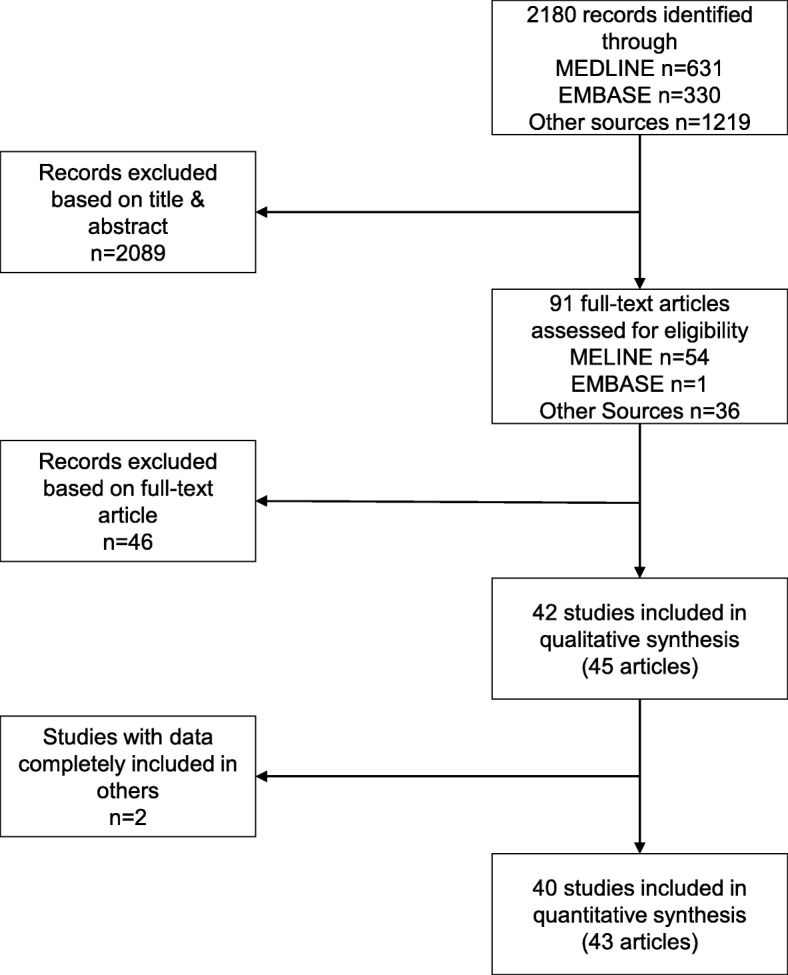
Flow diagram of study selection

We identified 40 eligible studies reported in 43 articles [21, 22, 32–72]. Figure 1 shows the selection process. We screened 2180 records and 91 full-text articles. The most common reason for excluding articles was that the assessment of trial registration was based solely on mention of a trial registration number in the article (Additional file 3: Table S1). In addition, we excluded two articles [73, 74] because the data were completely included in two other, larger studies [34, 35]. Finally, for one study [37], we excluded data for a subset of RCTs which were also covered in another study [35].

Table 1 shows the characteristics of included studies. The 40 studies covered a wide range of clinical specialties. To identify RCTs, 17 (43%) studies searched a sample of high-impact factor journals, while 10 (25%) studies performed a systematic review of RCTs on a specific clinical topic. Among 27 studies that identified RCTs based on publication in a specific sample of journals, the median number of included journals was 5 (Q1–Q3 4–10). Each study examined a median of 187 RCTs (Q1–Q3 103–301). Studies examined RCTs published over a median of 3 years (Q1–Q3 1–5 years), with a median starting year of 2009. To assess the registration status of published RCTs, all studies examined if a trial registration number was reported in the published article, except four studies. All studies except one searched trial registries, 28 (70%) searched ClinicalTrials.gov, and 29 (73%) searched the World Health Organization International Clinical Trials Registry Platform (WHO ICTRP). Finally, 19 (48%) studies contacted trial investigators to inquire about registration status. In all, 18 (45%) studies used all three methods (reporting of registration number, search in registries, and contact of authors).

**Table 1:** Characteristics of included studies

Reference	Medical specialty	Time period for RCT publication	Identification of trials	Journals (*N*)	Trials (*N*)	Assessment of registration			Registries searched				
						Reporting	Search	Email	CT.gov	ISRCTN	WHO ICTRP	ANZCTR	National registries
Ostervig [59]	Anesthesiology	2009–2014	Single journal	1	200	Yes	Yes	No	Yes	Yes	No	No	Yes
Jones [49]	Anesthesiology	2007; 2010; 2013; 2010	Selection of high IF journals	6	860	Yes	Yes	Yes	Yes	Yes	Yes	No	No
Nankervis [56]	Atopic dermatitis	01/2007–07/2011	SR of eczema RCTs	–	109	Yes	Yes	No	No	No	Yes	No	No
Mathieu [22]	Cardiology, rheumatology, gastroenterology	2008	SR of RCTs in cardiology, rheumatology, and gastroenterology	22	323	Yes	Yes	Yes	Yes	Yes	Yes	No	Yes
Wiebe [71]	Cardio-thoracic surgery	2008–2015	Selection of high IF journals	4	287	Yes	Yes	Yes	Yes	Yes	Yes	No	Yes
Emdin [42, 58]	Cardiovascular disease	12/2012	SR of cardiovascular disease RCTs	–	191	Yes	Yes	No	No	No	Yes	No	No
Cybulski [37]	Clinical psychology	2013	Selection of high IF journals	25	101	Yes	Yes	Yes	Yes	No	Yes	No	No
Anand [32]	Critical care	01/2005–08/2011	SR of RCTs in critical care	–	90	Yes	Yes	Yes	Yes	No	No	Yes	No
Shinohara [66]	Depression	2011–2013	SR of RCTs of cognitive behavioral therapy and new-generation antidepressants	–	170	Yes	No	Yes	Unclear				
Jones [48]	Emergency medicine	2008–2011	Selection of high IF journals	5	123	Yes	Yes	No	Yes	Yes	Yes	No	No
Farquhar [43, 44]	Fertility medicine	2010–2014	SR of RCTs of fertility treatments	NA	693	Yes	Yes	Yes	Yes	No	Yes	No	No
Li [52]	Gastroenterology and hepatology	2009–2012	Selection of high IF journals	10	305	Yes	Yes	No	Yes	Yes	Yes	Yes	No
Mann [53]	Geriatrics	2008–2012	Selection of high IF journals	5	220	Yes	Yes	No	No	No	Yes	No	No
McGee [54]	Kidney transplantation	10/2005–12/2010	SR of kidney transplantation RCTs	–	307	No	Yes	No	No	No	Yes	No	No
Gray [46]	Nursing	11/2011–09/2016	Key general and mental health journals in nursing	3	135	Yes	Yes	Yes	Yes	Yes	No	No	Yes
Byrne [36]	Obesity	01/2011–06/2016	Selection of specialty journals	4	223	Yes	Yes	Yes	Yes	Yes	Yes	Yes	Yes
You [33, 72]	Oncology	2005–2009	Journals thought to publish majority of oncology RCTs	10	366	Yes	Yes	No	Yes	Yes	No	No	No
Smail-Faugeron [68]	Oral health	2013	Selection of high IF journals	15	317	Yes	Yes	No	No	No	Yes	No	No
Hamm [21]	Pediatrics	2007	SR of pediatric RCTs	–	300	Yes	Yes	No	Yes	Yes	Yes	No	Yes
Gates [45]	Pediatrics	2012	SR of pediatric RCTs	–	300	Yes	Yes	No	Yes	Yes	No	No	No
Rosati [64]	Pediatrics	07/2013–11/2013	Single journal	1	20	Yes	Yes	No	Yes	Yes	No	Yes	Yes
Pinto [61]	Physical therapy	2009	Indexed in PEDro	–	200	Yes	Yes	Yes	Yes	Yes	Yes	Yes	Yes
Pidgeon [60]	Plastic surgery	04/2014–03/2015	Selection of high IF journals	3	24	No	Yes	No	Yes	Yes	Yes	No	No
Scott [65]	Psychiatry	2009–2013	Selection of high IF journals	5	181	Yes	Yes	Yes	Yes	Yes	No	No	Yes
Milette [55]	Psychosomatic and behavioral medicine	01/2008–09/2009	Selection of high IF journals	4	63	Yes	Yes	Yes	Yes	Yes	Yes	No	Yes
Riehm [63]	Psychosomatic and behavioral medicine	01/2013–10/2014	Selection of high IF journals	4	76	Yes	Yes	Yes	Yes	Yes	Yes	No	Yes
Bradley [35]	Psychotherapy	2010–2014	Selection of high IF journals	5	112	Yes	Yes	Yes	Yes	Yes	Yes	No	Yes
Sims [67]	Shoulder arthroplasty	2005–2015	SR of shoulder arthroplasty RCTs	10	37	Yes	Yes	Yes	Yes	Yes	Yes	No	No
Killeen [50]	Surgery	2009–2010	Selection of high IF journals	10	246	Yes	Yes	Yes	Yes	Yes	Yes	No	Yes
Hardt [47]	Surgery	06/2012–12/2012	Selection of high IF journals	10	103	Yes	Yes	No	No	No	Yes	No	No
Kunath [51]	Urology	2009	Selection of high IF journals	10	106	Yes	Yes	No	No	No	Yes	No	No
*Potentially overlapping studies*													
Bonnot [34]	Anesthesiology	2013	Selection of high IF journals	12	183	Yes	Yes	Yes	No	No	Yes	No	No
El-Boghdadly [41]	Anesthesiology	01/201412/2016	Single journal	1	90	Yes	Yes	No	Yes	Yes	No	Yes	Yes
Norris [57]	Diabetes, osteoporosis, lip-modifying agents	2005–2010	Comparative effectiveness reviews	–	299	No	Yes	No	No	No	Yes	No	No
Dekkers [40]	Multiple	2004–2012	Trial protocols submitted to ethics committee	–	54	Yes	Yes	No	No	No	Yes	No	No
Dechartres [38]	Multiple	2006–2014	Cochrane reviews	–	322	Yes	Yes	Yes	Yes	No	Yes	No	No
Dekkers [39]	Multiple	02/2009	SR of non-inferiority RCTs in Core Clinical Journals	121	133	Yes	Yes	No	Yes	Yes	No	No	No
Reveiz [62]	Multiple	2010	SR of RCTs from Latin America and Caribbean	–	526	No	Yes	No	No	No	Yes	No	No
Van de Wetering [70]	Multiple	11/2010	Core Clinical Journals	121	302	Yes	Yes	Yes	Yes	Yes	Yes	No	No
Walker [69]	Multiple	05/2011–05/2012	Selection of high IF journals	2	76	Yes	Yes	No	Yes	Yes	No	No	Yes

*SR* systematic review, *RCT* randomized controlled trial, *IF* impact factor, *PEDro* Physiotherapy Evidence Database, *CT.gov* ClinicalTrials.gov, *ISCRCTN* International Standard Randomised Controlled Trials Number, *WHO ICTRP* World Health Organization International Clinical Trials Registry Platform, *ANZCTR* Australian New Zealand Clinical Trials Registry

### Prevalence of registration

**Fig. 2:**
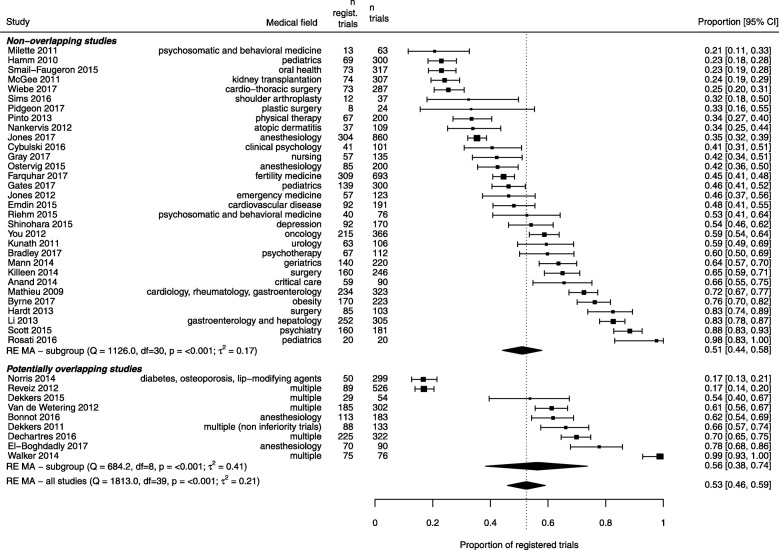
Combined estimates of the prevalence of trial registration

We included 31 non-overlapping studies in the main analysis. The studies examined a total of 6788 RCTs, among which 3267 (48%) RCTs had been registered. The proportion of registered trials varied considerably across studies, ranging from 21 to 100% (*Q* statistic 1126, df = 30, *p* < 0.001, between-study variance 0.17). In a random-effects meta-analysis, the pooled proportion of registered RCTs was 51% (95% confidence interval 44 to 58%) (Fig. 2). In a secondary analysis, we included an additional nine studies examining 1985 RCTs, which potentially overlapped in part with the primary sample. Among these, 924 (47%) RCTs were registered. When combining all 40 studies, totaling 8773 RCTs, the pooled proportion of registered RCTs was 53% (95% confidence 46 to 59%) with considerable between-study heterogeneity. A funnel plot did not show evidence of small-study effects (Additional file 3: Figure S1).

**Fig. 3:**
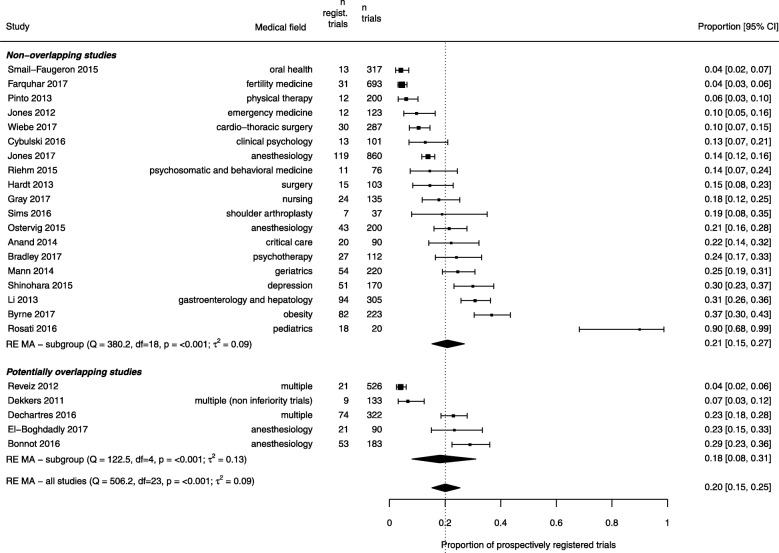
Combined estimates of the prevalence of prospective trial registration

Among the 31 non-overlapping studies, 19 also reported the number of trials that were registered prospectively. The studies included a total of 4272 RCTs, among which 676 (16%) RCTs were registered prospectively. There was a considerable heterogeneity across studies, with the proportion of prospectively registered RCTs ranging from 4 to 90% (*Q* statistic 380, df = 18, *p* < 0.001, between-study variance 0.09). The pooled proportion of prospectively registered RCTs was 21% (95% confidence interval 15 to 27%) (Fig. 3). Of the additional nine potentially overlapping studies included in the secondary analysis, five examined prospective registration across 1257 RCTs. When combining all 24 studies totaling 5529 RCTs, the pooled proportion of prospectively registered RCTs was 20% (95% confidence interval 15 to 25%). In addition, across these 24 studies, 1734 (67%) RCTs among 2588 registered RCTs were registered retrospectively, for a pooled proportion of 65% (95% confidence interval 59 to 71%).

### Prevalence of registration over time in study subgroups

Four studies [32, 35, 65, 66] included only studies that started to enroll participants exclusively after 2005, and another four studies [38, 47, 51, 59] included sub-analyses based on studies enrolling only after 2005. Across these eight studies totaling 938 RCTs, 622 (66%) were registered, and the pooled proportion was 65% (95% confidence interval 50 to 78%) (Additional file 3: Figure S2).

**Fig. 4:**
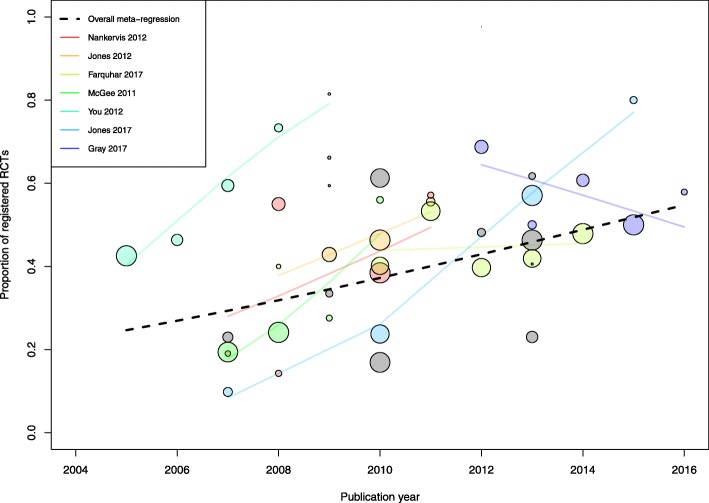
Meta-regression analysis of the prevalence of trial registration in relation to publication year. Each circle represents one study, and the size of each circle represents the weight given to the study in meta-regression. Separate meta-regression models were fitted in 7 studies that reported trial registration data by publication year. The black dashed line corresponds to an overall meta-regression model across these 7 studies with 11 studies that examined RCTs published in a single year. It showed that the proportion of registered trials increased over time, from 23% in 2005 to 52% in 2015 (29% increase, *p* = 0.03)

Seven studies reported data on registration according to RCT publication year (Fig. 4). Separate meta-regression models showed that the proportion of registered trials increased over time in five of these studies. Moreover, 12 studies examined RCTs published in a single year (*n* = 1 for 2007 [21], *n* = 3 for 2009 [39, 51, 61], *n* = 2 for 2010 [62, 70], *n* = 2 for 2012 [42, 45]; *n* = 4 for 2013 [34, 37, 64, 68]) (Additional file 3: Table S2). When combining all 19 studies, totaling 5144 RCTs, a meta-regression model showed that the proportion of registered trials increased significantly over time (*p* = 0.03), with a mean absolute proportion increase of 27% between 2005 and 2015, from 25 to 52%. In addition, 7 of the 12 studies that examined RCTs published in a single year reported data on prospective registration (Additional file 3: Table S2). A meta-regression model suggested that the proportion of prospectively registered trials increased as well, from 3% in 2009 to 21% in 2013 (18% increase, *p* = 0.04) (Additional file 3: Figure S3). Finally, in an analysis limited to studies published in 2010 or after, 26 studies reported data on 5401 RCTs. Of these, 2550 (47%) were registered, for a pooled proportion of 54% (95% confidence interval 47 to 60%).

### Prevalence of registration based on certain trial features

Nine studies examined registration of published RCTs according to industry funding. Across the nine studies, 778 of 2306 RCTs (34%) were supported fully or partially by industry sources. Of the 778 RCTs, 475 (61%) were registered. The pooled proportion was 59% (95% confidence interval 47 to 71%), as compared to 43% (95% confidence interval 30 to 58%) among trials not supported by industry. Five studies examined the prevalence of registration according to the trial sample size (Table 2). In all studies, there was evidence of higher registration prevalence among larger RCTs. Finally, 17 studies identified published RCTs from high-impact factor journals only. Out of 3383 RCTs, 1724 (51%) were registered. The pooled proportion was 55% (95% confidence interval 44 to 66%) as compared to 50% (95% confidence interval 42 to 59%) in the other 23 studies (5390 RCTs).

**Table 2:** Prevalence of registration among published randomized trials according to trial size

Reference	Sample size	Number of trials	Number of registered trials	Proportion of registered trials (95% CI)	*p* value*
Jones [48]	< 100	48	17	0.35 (0.22; 0.51)	0.009
	100–199	36	14	0.39 (0.23; 0.57)	
	200–499	30	21	0.70 (0.51; 0.85)	
	≥ 500	9	5	0.56 (0.21; 0.86)	
Mann [53]	< 100	71	33	0.46 (0.35; 0.59)	0.0009
	100–499	117	83	0.71 (0.62; 0.79)	
	500–999	13	10	0.77 (0.46; 0.95)	
	≥ 1000	15	12	0.80 (0.52; 0.96)	
McGee [54]	< 200	235	40	0.17 (0.12; 0.22)	< 0.0001
	≥ 200	72	34	0.47 (0.35; 0.59)	
Pinto [61]	≤ 25	34	5	0.15 (0.05; 0.31)	< 0.0001
	26–50	53	13	0.25 (0.14; 0.38)	
	51–100	61	19	0.31 (0.20; 0.44)	
	101–499	42	23	0.55 (0.39; 0.70)	
	≥ 500	10	7	0.70 (0.35; 0.93)	
Reveiz [62]	≤ 100	442	59	0.13 (0.10; 0.17)	< 0.0001
	> 100	84	30	0.36 (0.26; 0.47)	

*Chi-squared test for trend in proportions in studies with three or more categories or chi-squared test for studies with two categories

## Discussion

In this systematic review, we found that, among published RCTs, the proportion of registered and prospectively registered RCTs has increased over time but lack of registration and retrospective registration are still common. In analyses of more than 8000 RCTs published in medical journals, half of the RCTs published in recent years had not been registered, and 4 in 5 published RCTs had not been registered prospectively. Registration prevalence was higher in trials supported by industry funding, in larger trials, and in trials published in high-impact factor journals. While sharing of individual participant data has recently garnered much attention, our findings highlight the need for renewed efforts to address the first step on the continuum of research transparency and make prospective trial registration a top priority. Without prospective registration, our ability to monitor and resolve issues in trial reporting is substantially diminished.

Our synthesis shows that publication of unregistered trials and of trials registered retrospectively in medical journals persists. Many journals do not endorse the ICMJE registration policy and continue to support the publication of unregistered trials [75, 76]. In a mixed-methods analysis, editors and publishers reported several reasons for why journals do not reject unregistered or retrospectively registered articles, including concerns about losing submissions or preventing publication of studies from developing countries. Conducting trials without making all the results publicly available is unethical [77, 78]. Publishing the results of unregistered trials may be considered an ethical imperative as it does ensure that the research community has access to the results of these trials. But the large prevalence of unregistered trials published in medical journals raises concerns about persistent lack of transparency, underreporting or misreporting of trials, and biases in the resulting scientific literature. It directly undermines the first key objective of registries, which is to form a public “denominator” of all initiated trials, so that trials left unpublished can be identified and the available evidence interpreted in the context of unreported trials [79, 80].

Moreover, permitting publication of retrospectively registered trials defeats the second key objective of trial registration, which is to provide timestamped amendments to trial protocols. If a trial is registered after the enrollment of the first participants, it is no longer possible to compare reported results to the original trial record in order to identify selective reporting of outcomes and analyses [81, 82]. Among published RCTs that were registered, we found that 65% had been registered retrospectively. Zarin et al. [18] found a lower proportion; among 49 751 RCTs registered in ClinicalTrials.gov between 2012 and 2014, 33% had been registered more than 3 months after the trial start. Possible reasons for the difference is that our results concern only published RCTs; in contrast, most registered trials on ClinicalTrials.gov do not report results in a timely fashion [19]. In addition, we examined studies that assessed prospective registration according to the ICMJE or FDAAA definitions, which stipulates registration prior to the onset of patient enrollment or no later than 21 days after enrollment of the first participant. A higher proportion of trials may have been classified as prospectively registered had the cutoff been 3 months. Another reason for the difference is that we included trials registered in other registries and that practices with regard to prospective registration might be different among trials registered in these. Lastly, among 123 trials rejected by the BMJ between June 2013 and June 2017 because they did not comply with ICMJE trial registration requirements, 89% were retrospectively registered and 7% were unregistered [83].

Our findings have implications for systematic reviewers. Roberts et al. have suggested that systematic reviews include only prospectively registered trials, under the premise that such trials are the only ones not affected by reporting bias. Registered and unregistered trials have been found to differ in their risk of bias in studies examining 326 RCTs from Latin America and the Caribbean and 693 RCTs of fertility treatments [44, 62]. Other investigations have examined the impact of registration status on positive study findings and have not found differences between registered and non-registered trials [38, 42, 58]. We do not endorse restricting a systematic review to only registered trials but, given the large number of unregistered trials in the current medical literature and the potential difference from registered trials, systematic review authors should conduct subgroup analyses in cases where both registered and unregistered trials contribute to a meta-analysis. Such analyses should ideally distinguish between unregistered trials, retrospectively registered trials, prospectively registered trials with potential outcome reporting bias, and prospectively registered trials with no outcome reporting bias.

Our findings also have implications for a range of stakeholder groups focused on improving trial registration. Many actions have already been implemented by journal editors, regulatory agencies, and funding organizations to tackle the lack of prospective registration [18]. Because of the inherent lag between registration and publication, we could see substantial changes in upcoming years in response to the Final Rule and the new National Institutes of Health policy. However, existing laws and policies may not be sufficient and novel interventions may be required to increase trial registration. Many organizations in the USA do not have policies, staff, or other resources needed to ensure their trials are registered and reported in a timely fashion [84]. Twenty stakeholders have recently affirmed that prospective registration is of critical importance and that they will implement policies with monitoring systems to improve registration and reporting of results. In a recent commentary, Loder suggested treating unregistered or retrospectively registered trials as medical “never events.” Such events should trigger drastic responses, similar to specified events in clinical medicine. For example, Dr. Loder argues that journal editors and peer reviewers should verify that trial registration occurred before the trial enrolment began and, according to the ICMJE policy, reject trials registered retrospectively. If not published in medical journals, trial results could still be posted online. Most importantly, we believe that multiple entities, including funding agencies, ethics committees, and academic institutions should continue to enforce standards of universal trial registration [85–88]. For example, these stakeholders could take prospective registration into account when considering full grant payments or academic promotions.

Our systematic review has limitations. First, some included studies examined RCTs that started prior to 2005 when registration requirements were implemented. However, we conducted analyses limited to RCTs started after 2005 and to RCTs published after 2010 that suggest that the low prevalence of registration among published RCTs has persisted among recent trials. Second, we could not rule out the possibility that some study samples overlapped with others among the 40 included studies. However, our primary analysis was restricted to 31 non-overlapping studies. The secondary analysis, which included all 40 studies, showed the same average prevalence of registration as in the primary analysis. Third, we were not able to fully explore sources of variability in the prevalence of registration, though we found that trial registration varied substantially across clinical fields and journals. Moreover, registration prevalence was higher among trials supported by the industry, larger trials, and trials in high-impact factor journals. Data were not available on trial location, and compliance with trial registration is likely to vary across countries. Viergever and Li [14] have shown that trends in registration on WHO ICTRP did not take place equally in all parts of the world. Fourth, our results apply to RCTs, while registration requirements apply broadly to all types of clinical trials. We therefore cannot ascertain whether the prevalence of registration we report here would be the same across all clinical study designs. Fifth, the included studies used different methods to ascertain whether published RCTs were registered. Some included studies might have missed registered RCTs and thus possibly underestimate the proportion of registered RCTs. Conversely, we excluded studies that assessed trial registration based solely on the mention of a trial registration number in the article, because such studies would underestimate the proportion of registered RCTs. However, trial registration is useful if end users can identify trial records. In this regard, the proportion of registered RCTs we found might be larger than the proportion of “useful” registrations. Sixth, data on the proportion of prospectively registered RCTs according to publication year were limited. Finally, in our systematic review, we have not assessed the quality of the registration of outcomes. The lack of a detailed specification of outcomes may also introduce reporting biases [89].

## Conclusions

Non-registration and retrospective registration of clinical trials remain common, undermining the validity and integrity of biomedical research. Given long-standing policies mandating registration, enforcing prospective registration will likely require novel interventions and greater endorsement by a range of stakeholders in the research community, including investigators, funding entities, ethical oversight bodies, and journal editors. Universal, prospective trial registration should be a top priority in the endeavors to improve research transparency and ensure rigorous, high-quality evidence is available to inform patient care.

## Additional files

Additional file 1: abstracted data. (CSV 2 kb)

Additional file 2: R code. (HTML 1110 kb)

Additional file 3: PRISMA checklist. (DOCX 149 kb)

## Abbreviations

ANZCTR: Australian New Zealand Clinical Trials Registry
CT.gov: ClinicalTrials.gov
FDA: Food and Drug Administration
FDAAA: Food and Drug Administration Amendments Act
ICMJE: International Committee of Medical Journal Editors
ISCRCTN: International Standard Randomised Controlled Trials Number
IF: impact factor
PEDro: Physiotherapy Evidence Database
RCT: randomized controlled trial
SR: systematic review
WHO ICTRP: World Health Organization International Clinical Trials Registry Platform
