# Supplementary material for: Registration of published randomized trials: a systematic review and meta-analysis
Source: BMC Med. 2018 Oct 16;16:173. doi: 10.1186/s12916-018-1168-6 (PMC6190546; doi:10.1186/s12916-018-1168-6)
Supplement: Supplementary file 2 — R code. (HTML 1110 kb) [file 12916_2018_1168_MOESM2_ESM.html]

Registration of published randomized trials: a systematic review and meta-analysis


# Registration of published randomized trials: a systematic review and meta-analysis

#### *L Trinquart, A Dunn, F Bourgeois*

#### *Aug 2, 2018*

#### Meta-analysis function from Bakbergenuly Biom J 2016

```
library(metafor)
```

```
## Warning: package 'metafor' was built under R version 3.4.4
```

```
## Loading required package: Matrix
```

```
## Loading 'metafor' package (version 2.0-0). For an overview 
## and introduction to the package please type: help(metafor).
```

```
rma.arcsine<-function(ai,ni){
   k         <-length(ai)
   p         <-ai/ni
   ##ARCSIN transformation
   ###inverse variance with fixed effects model
   hIV       <-2*asin(sqrt(p))
   varIV     <-1/(ni)
   wiIV      <-1/varIV
   errorPIV    <-1/2*sqrt(sum(wiIV)^-1)*qnorm(0.975)
   h_barIV   <-sum(wiIV*hIV)/sum(wiIV)
   ###inverse variance with random effects model for arcsin
   pIV       <-(sin(h_barIV/2))^2
   pIV_U     <-(sin(h_barIV/2+errorPIV))^2
   pIV_L     <-(sin(h_barIV/2-errorPIV))^2
   
   hREM      <-2*asin(sqrt(p))
   varREM    <-1/(ni)
   ##DerSinomian-Laird method for tau
   w<-1/varREM
   W<-sum(w)
   hbar<-sum(hREM*w)/W
   Q<-sum(w*(hREM-hbar)^2)
   S2<-sum(w^2)
   ###DerSimonian and Laird estimator of between study variance
   tau2<-max(0, (Q-k+1)/(W-S2/W))
   wiREM     <-1/(varREM+tau2)
   errorPREM <-1/2*sqrt(sum(wiREM)^-1)*qnorm(0.975)   
   h_barREM  <-sum(wiREM*hREM)/sum(wiREM)
   pREM      <-(sin(h_barREM/2))^2
   pREM_U    <-(sin(h_barREM/2+errorPREM))^2
   pREM_L    <-(sin(h_barREM/2-errorPREM))^2
   
  
   ###Assuming beta-binomial model and using arcsin
   ###estimation of intra-cluster correlation 
   N         <-sum(ni)
   MS_B      <-1/(k-1)*(sum(ai^2/ni)-1/N*(sum(ai))^2)
   MS_w      <-1/(N-k)*(sum(ai)-sum(ai^2/ni))
   n0        <-(N-sum((ni^2)/N))/(k-1)
   rho       <-(MS_B-MS_w)/(MS_B+(n0-1)*MS_w)
   
   ##################################
   wiBB      <-ni/(1+rho*(ni-1))#weights
   errorPBB  <-1/2*sqrt(sum(wiBB)^-1)*qnorm(0.975)  
   #without continuity correction
   h0BB        <-2*asin(sqrt(p))
   h0_barBB  <-sum(wiBB*h0BB)/sum(wiBB)
   p0BB      <-(sin(h0_barBB/2))^2
   p0BB_U    <-(sin(h0_barBB/2+errorPBB))^2
   p0BB_L    <-(sin(h0_barBB/2-errorPBB))^2
   #without continuity correction
   p1        <-(ai+3/8)/(ni+3/4)
   hBB         <-2*asin(sqrt(p1))
   h_barBB   <-sum(wiBB*hBB)/sum(wiBB)
   pBB       <-(sin(h_barBB/2))^2
   pBB_U     <-(sin(h_barBB/2+errorPBB))^2
   pBB_L     <-(sin(h_barBB/2-errorPBB))^2
   
   
   ######Correcting the bias in arcsin transformation
   pcc     <-(ai+0.5)/(ni+1)  ##estimation of probability with standard continuity correction
   p0      <-(ai/ni)
   h0      <-2*asin(sqrt(p0))+((1-2*pcc)/sqrt(pcc*(1-pcc))*(1+(ni-1)*rho)/4/ni)
   h0_barBiasCor  <-sum(wiBB*h0)/sum(wiBB)
   p0BiasCor      <-(sin(h0_barBiasCor/2))^2
   p0UBiasCor     <-(sin(h0_barBiasCor/2+errorPBB))^2
   p0LBiasCor     <-(sin(h0_barBiasCor/2-errorPBB))^2
   
   #without continuity correction
   p1      <-(ai+3/8)/(ni+3/4)
   h       <-2*asin(sqrt(p1))-(3*(1-2*pcc)/(2*sqrt(pcc*(1-pcc))*(4*ni+3))-(1-2*pcc)*4*ni*(1+(ni-1)*rho)/(sqrt(pcc*(1-pcc))*(4*ni+3)^2))
   h_barBiasCor   <-sum(wiBB*h)/sum(wiBB)
   pBiasCor       <-(sin(h_barBiasCor/2))^2
   pUBiasCor      <-(sin(h_barBiasCor/2+errorPBB))^2
   pLBiasCor      <-(sin(h_barBiasCor/2-errorPBB))^2
   return(cbind(pIV,pIV_L,pIV_U,pREM,pREM_L,pREM_U,p0BB,p0BB_L,p0BB_U,pBB,pBB_L,pBB_U,p0BiasCor,p0LBiasCor,p0UBiasCor,pBiasCor,pLBiasCor,pUBiasCor,rho,tau2,Q))
   }
```

#### Importing data

```
ds<-read.table("C:\\Users\\ludovic\\Dropbox\\SR registration\\data & analysis\\data20180305.csv",sep=",",header=TRUE, stringsAsFactors = FALSE)
ds$id <- seq.int(nrow(ds))
```

#### Prevalence of registration

```
overall<-rma.arcsine(ds$n.reg.trials,ds$n.trials)
no.overlap<-rma.arcsine(ds[ds$group==0,]$n.reg.trials,ds[ds$group==0,]$n.trials)
overlap<-rma.arcsine(ds[ds$group==2,]$n.reg.trials,ds[ds$group==2,]$n.trials)

ds<-escalc(xi=n.reg.trials,ni=n.trials, data=ds, measure ="PR",append = TRUE)

tmp <- t(sapply(split(ds, ds$id), function(x) binom.test(x$n.reg.trials, x$n.trials)$conf.int))
ds$ci.lb <- tmp[,1]
ds$ci.ub <- tmp[,2]

op<-par(mar=c(3.5,2.5,1.5,2.5))
par(op)
with(ds, forest(yi,ci.lb=ci.lb, ci.ub=ci.ub, ylim=c(-0.5,48), xlim=c(-1.3,1.4), refline=overall[16],subset=order(-ds$group,-ds$yi),cex=0.75,
slab=ds$author,ilab=cbind(ds$med.field, ds$n.reg.trials, ds$n.trials),
ilab.xpos=c(-0.16,-0.05,0.07),ilab.pos=c(2,2,2), rows=c(2:10,14:44),xlab="Proportion of registered trials"))

addpoly(x=overall[16],ci.lb=overall[17],ci.ub=overall[18],cex=0.75)
addpoly(x=overlap[16],ci.lb=overlap[17],ci.ub=overlap[18],cex=0.75, row=1)
addpoly(x=no.overlap[16],ci.lb=no.overlap[17],ci.ub=no.overlap[18],cex=0.75, row=13)

par(font=4)
text(-1.28, 45, "Non-overlapping studies", cex=0.75,adj = c(0,NA) )
text(-1.28, 11, "Potentially overlapping studies", cex=0.75,adj = c(0,NA) )
par(font=1)

text(-1.28, 47, "Study", cex=0.75,adj = c(0,NA) )
text(-0.2, 47, "Medical field", cex=0.75, adj=c(1,NA))
text(-0.06, 48, "n \nregist.\ntrials", cex=0.75, adj=c(1,NA))
text(-0.02, 48, "n \ntrials", cex=0.75, adj=c(0,NA))
text(1.4,   47, "Proportion [95% CI]",cex=0.75, adj=c(1,NA))

text(-1.28, 13, adj=c(0,NA), cex=0.75, bquote(paste("RE MA - subgroup (Q = ",
.(formatC(no.overlap[21], digits=1, format="f")), ", df=", .(dim(ds[ds$group==0,])-1),
", p = ", .(format.pval(pchisq(no.overlap[21],dim(ds[ds$group==0,])[1]-1,lower.tail=FALSE), eps = .001, digits = 2)), "; ", tau^2, " = ",
.(formatC(no.overlap[20], digits=2, format="f")),")")))

text(-1.28, 1, adj=c(0,NA), cex=0.75, bquote(paste("RE MA - subgroup (Q = ",
.(formatC(overlap[21], digits=1, format="f")), ", df=", .(dim(ds[ds$group==2,])-1),
", p = ", .(format.pval(pchisq(overlap[21],dim(ds[ds$group==2,])[1]-1,lower.tail=FALSE), eps = .001, digits = 2)), "; ", tau^2, " = ",
.(formatC(overlap[20], digits=2, format="f")),")")))

text(-1.28, -0.5, adj=c(0,NA), cex=0.75, bquote(paste("RE MA - all studies (Q = ",
  .(formatC(overall[21], digits=1, format="f")), ", df=", .(dim(ds)-1),
  ", p = ", .(format.pval(pchisq(overall[21],dim(ds)[1]-1,lower.tail=FALSE), eps = .001, digits = 2)), "; ", tau^2, " = ",
  .(formatC(overall[20], digits=2, format="f")),")")))
```

#### Funnel plot

```
ds.fp<-escalc(xi=n.reg.trials,ni=n.trials, data=ds, measure ="PLO",append = TRUE)
funnel(ds.fp$yi, ds.fp$vi, back="white")
```

#### Prospective registration

```
ds.prosp<-ds[!is.na(ds$n.trials.reg.prosp),]
ds.prosp<-escalc(xi=n.trials.reg.prosp,ni=n.trials2, data=ds.prosp, measure ="PR",append = TRUE)

tmp <- t(sapply(split(ds.prosp, ds.prosp$id), function(x) binom.test(x$n.trials.reg.prosp, x$n.trials)$conf.int))
ds.prosp$ci.lb <- tmp[,1]
ds.prosp$ci.ub <- tmp[,2]

overall.prosp<-rma.arcsine(ds.prosp$n.trials.reg.prosp,ds.prosp$n.trials)
no.overlap.prosp<-rma.arcsine(ds.prosp[ds.prosp$group==0,]$n.trials.reg.prosp,ds.prosp[ds.prosp$group==0,]$n.trials)
overlap.prosp<-rma.arcsine(ds.prosp[ds.prosp$group==2,]$n.trials.reg.prosp,ds.prosp[ds.prosp$group==2,]$n.trials)

op<-par(mar=c(3.5,2.5,1.5,2.5))
par(op)
with(ds.prosp, forest(yi,ci.lb=ci.lb, ci.ub=ci.ub, ylim=c(-0.5,32), xlim=c(-1.4,1.4), refline=overall.prosp[16],subset=order(-ds.prosp$group,-ds.prosp$yi),cex=0.75,
slab=ds.prosp$author,ilab=cbind(ds.prosp$med.field, ds.prosp$n.trials.reg.prosp, ds.prosp$n.trials),
ilab.xpos=c(-0.25,-0.12,0.0),ilab.pos=c(2,2,2), rows=c(2:6,10:28),xlab="Proportion of prospectively registered trials"))

addpoly(x=overall.prosp[16],ci.lb=overall.prosp[17],ci.ub=overall.prosp[18],cex=0.75)
addpoly(x=overlap.prosp[16],ci.lb=overlap.prosp[17],ci.ub=overlap.prosp[18],cex=0.75, row=1)
addpoly(x=no.overlap.prosp[16],ci.lb=no.overlap.prosp[17],ci.ub=no.overlap.prosp[18],cex=0.75, row=9)

par(font=4)
text(-1.38, 29, "Non-overlapping studies", cex=0.75,adj = c(0,NA) )
text(-1.38, 7, "Potentially overlapping studies", cex=0.75,adj = c(0,NA) )
par(font=1)

text(-1.38, 31, "Study", cex=0.75,adj = c(0,NA) )
text(-0.3, 31, "Medical field", cex=0.75, adj=c(1,NA))
text(-0.15, 32, "n \nregist.\ntrials", cex=0.75, adj=c(1,NA))
text(-0.1, 32, "n \ntrials", cex=0.75, adj=c(0,NA))
text(1.4,   31, "Proportion [95% CI]",cex=0.75, adj=c(1,NA))

text(-1.38, 9, adj=c(0,NA), cex=0.75, bquote(paste("RE MA - subgroup (Q = ",
.(formatC(no.overlap.prosp[21], digits=1, format="f")), ", df=", .(dim(ds.prosp[ds.prosp$group==0,])-1),
", p = ", .(format.pval(pchisq(no.overlap.prosp[21],dim(ds.prosp[ds.prosp$group==0,])[1]-1,lower.tail=FALSE), eps = .001, digits = 2)), "; ", tau^2, " = ",
.(formatC(no.overlap.prosp[20], digits=2, format="f")),")")))

text(-1.38, 1, adj=c(0,NA), cex=0.75, bquote(paste("RE MA - subgroup (Q = ",
.(formatC(overlap.prosp[21], digits=1, format="f")), ", df=", .(dim(ds.prosp[ds.prosp$group==2,])-1),
", p = ", .(format.pval(pchisq(overlap.prosp[21],dim(ds.prosp[ds.prosp$group==2,])[1]-1,lower.tail=FALSE), eps = .001, digits = 2)), "; ", tau^2, " = ",
.(formatC(overlap.prosp[20], digits=2, format="f")),")")))

text(-1.38, -0.5, adj=c(0,NA), cex=0.75, bquote(paste("RE MA - all studies (Q = ",
.(formatC(overall.prosp[21], digits=1, format="f")), ", df=", .(dim(ds.prosp)-1),
", p = ", .(format.pval(pchisq(overall.prosp[21],dim(ds.prosp)[1]-1,lower.tail=FALSE), eps = .001, digits = 2)), "; ", tau^2, " = ",
.(formatC(overall.prosp[20], digits=2, format="f")),")")))
```

#### Funding

##### supported by the industry

```
ntrials.industry<-c(14,56,16,84,126,67,72,221,122)
nregistered<-c(11,37,9,54,103,24,30,158,49)
rma.arcsine(nregistered,ntrials.industry)
```

```
##           pIV     pIV_L     pIV_U      pREM    pREM_L    pREM_U      p0BB
## [1,] 0.616267 0.5818372 0.6501229 0.5967368 0.4713764 0.7159875 0.5969062
##         p0BB_L    p0BB_U       pBB     pBB_L     pBB_U p0BiasCor
## [1,] 0.4756446 0.7124374 0.5947874 0.4734886 0.7104812 0.5892912
##      p0LBiasCor p0UBiasCor  pBiasCor pLBiasCor pUBiasCor       rho
## [1,]  0.4679064  0.7053959 0.5894822 0.4681002 0.7055729 0.1209656
##           tau2        Q
## [1,] 0.1281331 91.23152
```

##### not supported by the industry

```
ntrials<-c(98,148,107,162,196,233,254,145,185)
nregist<-c(56,97,48,106,122,45,59,57,25)
rma.arcsine(nregist,ntrials)
```

```
##            pIV     pIV_L     pIV_U      pREM  pREM_L    pREM_U      p0BB
## [1,] 0.3925629 0.3682237 0.4171722 0.4248379 0.28096 0.5755443 0.4249157
##         p0BB_L    p0BB_U       pBB     pBB_L     pBB_U p0BiasCor
## [1,] 0.2887046 0.5672082 0.4252103 0.2889747 0.5675034 0.4340383
##      p0LBiasCor p0UBiasCor  pBiasCor pLBiasCor pUBiasCor       rho
## [1,]  0.2970917  0.5763275 0.4339853 0.2970428 0.5762746 0.1858085
##           tau2        Q
## [1,] 0.2080413 287.5368
```

#### Enrollment after 2005

```
s<-c("Ostervig 2015", "Scott 2015", "Anand 2014", "Shinohara 2015","Bradley 2017", "Hardt 2013", "Kunath 2011", "Dechartres 2016")
n<-c(127,181,90,170,112,89,36,133)
r<-c(42,160,59,92,67,71,20,111)

ds.2005<-data.frame(s,n,r)
ds.2005<-escalc(xi=r,ni=n, data=ds.2005, measure ="PR",append = TRUE)
ds.2005$id<-1:nrow(ds.2005)

tmp <- t(sapply(split(ds.2005, ds.2005$id), function(x) binom.test(x$r, x$n)$conf.int))
ds.2005$ci.lb <- tmp[,1]
ds.2005$ci.ub <- tmp[,2]

overall.2005<-rma.arcsine(ds.2005$r,ds.2005$n)

op<-par(mar=c(3.5,2.5,1.5,2.5))
par(op)
with(ds.2005, forest(yi,ci.lb=ci.lb, ci.ub=ci.ub, ylim=c(-0.5,12), xlim=c(-1.0,1.4), refline=overall.2005[16],subset=order(-ds.2005$yi),cex=0.75,
slab=ds.2005$s,ilab=cbind(ds.2005$r, ds.2005$n),
ilab.xpos=c(-0.25,-0.12),ilab.pos=c(2,2), rows=c(2:9),xlab="Proportion of registered trials"))

addpoly(x=overall.2005[16],ci.lb=overall.2005[17],ci.ub=overall.2005[18],cex=0.75, row=1)

text(-1.0, 11, "Study", cex=0.75,adj = c(0,NA) )
text(-0.27, 11, "n \nregist.\ntrials", cex=0.75, adj=c(1,NA))
text(-0.22, 11, "n \ntrials", cex=0.75, adj=c(0,NA))
text(1.4,   11, "Proportion [95% CI]",cex=0.75, adj=c(1,NA))


text(-1.0, 1, adj=c(0,NA), cex=0.75, bquote(paste("RE MA (Q = ",
   .(formatC(overall.2005[21], digits=1, format="f")), ", df=", .(dim(ds.2005)-1),
   ", p = ", .(format.pval(pchisq(overall.2005[21],dim(ds.2005)[1]-1,lower.tail=FALSE), eps = .001, digits = 2)), "; ", tau^2, " = ",
   .(formatC(overall.2005[20], digits=2, format="f")),")")))
```

##### Publication year

```
library(scales)

n<-c(32, 22, 28, 34, 19, 316, 219, 170, 155, 94, 69, 74, 75, 54, 67, 58, 29, 25, 137, 137, 141, 136, 142, 20, 35, 41, 27, 21, 28, 20, 26, 14, 300, 200, 106, 526, 302, 191, 183, 101, 317, 133, 20, 300)
r<-c(22, 11, 17, 17, 11, 31, 52, 97, 124, 40, 32, 44, 55, 44, 13, 14, 8, 14, 55, 73, 56, 57, 68, 8, 15, 19, 15, 4, 4, 11, 10, 8, 69, 67, 63, 89, 185, 92, 113, 41, 73, 88, 20, 139)
y<-c(2012, 2013, 2014,2015,2016, 2007, 2010, 2013, 2015, 2005, 2006, 2007, 2008, 2009, 2007, 2008, 2009, 2010, 2010, 2011, 2012, 2013, 2014, 2008, 2009, 2010, 2011, 2007, 2008, 2008, 2010, 2011, 2007, 2009, 2009, 2010, 2010, 2012, 2013, 2013, 2013, 2009, 2012, 2013)
id<-c(7, 7, 7, 7, 7, 6, 6, 6, 6, 5, 5, 5, 5, 5, 4, 4, 4, 4, 3, 3, 3, 3, 3, 2, 2, 2, 2, 1, 1, 1, 1, 1, 8, 9, 10, 11, 12, 13, 14, 15, 16, 17, 18, 19)
ddsy<-data.frame(n,r,y,id)
ddsy<-escalc(xi=r,ni=n, data=ddsy, measure ="PLO",append = TRUE)

par(mfrow=c(1,1))

plot(1, type="n", xlab="Publication year", ylab="Proportion of registered RCTs", xlim=c(2004,2016), ylim=c(0,1))

for (i in 1:7){
model <- lm(ddsy[ddsy$id==i,]$yi ~ ddsy[ddsy$id==i,]$y, weights=1/ddsy[ddsy$id==i,]$vi^2)
print(summary(model)$coefficients[2,4])
symbols(x=ddsy[ddsy$id==i,]$y, y=1/(1+exp(-ddsy[ddsy$id==i,]$yi)), circles=1/ddsy[ddsy$id==i,]$vi^2, inches=0.15, bg=alpha(rainbow(9)[i],0.25), add=TRUE)
#points(x=ddsy[ddsy$id==i,]$y, y=1/(1+exp(-ddsy[ddsy$id==i,]$yi)), pch=15, col=terrain.colors(8)[i])
lines(sort(ddsy[ddsy$id==i,]$y), 1/(1+exp(-fitted(model)[order(ddsy[ddsy$id==i,]$y)])), col=alpha(rainbow(9)[i],0.25),lwd=2)
}
```

```
## [1] 0.4792892
## [1] 0.05962396
## [1] 0.8521786
## [1] 0.07581396
## [1] 0.004690971
## [1] 0.005487949
## [1] 0.2375696
```

```
model <- lm(ddsy$yi ~ ddsy$y, weights=1/ddsy$vi^2)
summary(model)
```

```
## 
## Call:
## lm(formula = ddsy$yi ~ ddsy$y, weights = 1/ddsy$vi^2)
## 
## Weighted Residuals:
##    Min     1Q Median     3Q    Max 
## -79.04  -2.37   2.16  15.30  70.27 
## 
## Coefficients:
##               Estimate Std. Error t value Pr(>|t|)  
## (Intercept) -239.53206  106.52644  -2.249   0.0298 *
## ddsy$y         0.11891    0.05297   2.245   0.0301 *
## ---
## Signif. codes:  0 '***' 0.001 '**' 0.01 '*' 0.05 '.' 0.1 ' ' 1
## 
## Residual standard error: 24 on 42 degrees of freedom
## Multiple R-squared:  0.1071, Adjusted R-squared:  0.08586 
## F-statistic: 5.039 on 1 and 42 DF,  p-value: 0.03011
```

```
print(summary(model)$coefficients[2,4])
```

```
## [1] 0.03011492
```

```
lines(sort(ddsy$y), 1/(1+exp(-fitted(model)[order(ddsy$y)])), col=9,lwd=3,lty=2)
symbols(x=ddsy[!ddsy$id %in% 1:7,]$y, y=1/(1+exp(-ddsy[!ddsy$id %in% 1:7,]$yi)), circles=1/ddsy[!ddsy$id %in% 1:7,]$vi^2, inches=0.15, bg=alpha(1,0.25), add=TRUE)

legend("topleft", c("Overall meta-regression", "Nankervis 2012", "Jones 2012","Farquhar 2017", "McGee 2011", "You 2012", "Jones 2017", "Gray 2017") , 
 col=c("#000000",rainbow(9)[1],rainbow(9)[2],rainbow(9)[3],rainbow(9)[4],
 rainbow(9)[5],rainbow(9)[6],rainbow(9)[7]),lty=c(2,1,1,1,1,1,1,1), lwd=c(3,1,1,1,1,1,1,1), cex=0.75)
```

```
#Prospective registration 
n<-c(300,106,183,133,526,101,317,300,191,200,302,20)
#r<-c(69, 63, 113, 88, 89, 41, 73, 139, 92, 67, 185, 20)
y<-c(2007, 2009, 2013, 2009, 2010, 2013, 2013, 2012, 2012, 2009, 2010, 2013)
#prospective
r<-c(NA,NA,53,9,21,13,13,NA,NA,12,NA,18)

ds.yr<-data.frame(n,r,y)
ds.yr<-na.omit(ds.yr)
ds.yr<-escalc(xi=r,ni=n, data=ds.yr, measure ="PR",append = TRUE)
ds.yr$id<-1:nrow(ds.yr)

tmp <- t(sapply(split(ds.yr, ds.yr$id), function(x) binom.test(x$r, x$n)$conf.int))
ds.yr$ci.lb <- tmp[,1]
ds.yr$ci.ub <- tmp[,2]
rma.arcsine(r,n)
```

```
##      pIV pIV_L pIV_U pREM pREM_L pREM_U p0BB p0BB_L p0BB_U pBB pBB_L pBB_U
## [1,]  NA    NA    NA   NA     NA     NA   NA     NA     NA  NA    NA    NA
##      p0BiasCor p0LBiasCor p0UBiasCor pBiasCor pLBiasCor pUBiasCor rho tau2
## [1,]        NA         NA         NA       NA        NA        NA  NA   NA
##       Q
## [1,] NA
```

```
ddsy<-escalc(xi=r,ni=n, data=ds.yr, measure ="PLO",append = TRUE)
model <- lm(ddsy$yi ~ ddsy$y, weights=1/ddsy$vi^2)

plot(1, type="n", xlab="Publication year", ylab="Proportion of prospectively registered RCTs", xlim=c(2004,2016), ylim=c(0,1))
lines(sort(ddsy$y), 1/(1+exp(-fitted(model)[order(ddsy$y)])), col=9,lwd=3,lty=2)
symbols(x=ddsy$y, y=1/(1+exp(-ddsy$yi)), circles=1/ddsy$vi^2, inches=0.3, bg="red", add=TRUE)
```

```
ds.yr$p<-1/(1+exp(-ddsy$yi))
```

#### Published after 2010

```
n<-c(191,101,170,693,135,223,317,24,76,112,103,183,526,76,302,14,27,137,141,136,142,170,155,32,22,28,34,19,191,183,101,317, 20, 300)
r<-c(92,41,92,309,57,170,73,8,40,67,85,113,89,75,185,8,15,73,56,57,68,97,124,22,11,17,17,11,92,113,41,73, 20, 139)
rma.arcsine(r,n)
```

```
##           pIV     pIV_L     pIV_U      pREM    pREM_L    pREM_U      p0BB
## [1,] 0.472866 0.4595623 0.4861891 0.5486616 0.4757502 0.6205377 0.5482783
##         p0BB_L    p0BB_U       pBB     pBB_L     pBB_U p0BiasCor
## [1,] 0.4831542 0.6125825 0.5440639 0.4789248 0.6084548 0.5401783
##      p0LBiasCor p0UBiasCor  pBiasCor pLBiasCor pUBiasCor       rho
## [1,]  0.4750295  0.6046449 0.5385614 0.4734096 0.6030583 0.1377825
##           tau2        Q
## [1,] 0.1737857 921.1975
```

#### Trial size

```
n<-c(48,36,30,9,71,117,13,15,235,72,34,53,61,42,10,84,442)
r<-c(17,14,21,5,33,83,10,12,40,34,5,13,19,23,7,30,59)
id<-c(1,1,1,1,2,2,2,2,3,3,4,4,4,4,4,5,5)

for (i in 1:17){print(binom.test(r[i], n[i])$conf.int)}
```

```
## [1] 0.2216059 0.5054317
## attr(,"conf.level")
## [1] 0.95
## [1] 0.2314244 0.5653620
## attr(,"conf.level")
## [1] 0.95
## [1] 0.5060410 0.8526548
## attr(,"conf.level")
## [1] 0.95
## [1] 0.2120085 0.8630043
## attr(,"conf.level")
## [1] 0.95
## [1] 0.3454792 0.5871278
## attr(,"conf.level")
## [1] 0.95
## [1] 0.6182551 0.7896352
## attr(,"conf.level")
## [1] 0.95
## [1] 0.4618685 0.9496189
## attr(,"conf.level")
## [1] 0.95
## [1] 0.5191089 0.9566880
## attr(,"conf.level")
## [1] 0.95
## [1] 0.1244739 0.2244859
## attr(,"conf.level")
## [1] 0.95
## [1] 0.3533297 0.5934709
## attr(,"conf.level")
## [1] 0.95
## [1] 0.04952846 0.31056573
## attr(,"conf.level")
## [1] 0.95
## [1] 0.1375512 0.3828247
## attr(,"conf.level")
## [1] 0.95
## [1] 0.1990336 0.4429366
## attr(,"conf.level")
## [1] 0.95
## [1] 0.3867318 0.7015412
## attr(,"conf.level")
## [1] 0.95
## [1] 0.3475471 0.9332605
## attr(,"conf.level")
## [1] 0.95
## [1] 0.2555143 0.4691631
## attr(,"conf.level")
## [1] 0.95
## [1] 0.1031903 0.1687838
## attr(,"conf.level")
## [1] 0.95
```

```
for (i in c(1,2,4)){print(  prop.trend.test(r[id==i], n[id==i]))}
```

```
## 
##  Chi-squared Test for Trend in Proportions
## 
## data:  r[id == i] out of n[id == i] ,
##  using scores: 1 2 3 4
## X-squared = 6.9142, df = 1, p-value = 0.008551
## 
## 
##  Chi-squared Test for Trend in Proportions
## 
## data:  r[id == i] out of n[id == i] ,
##  using scores: 1 2 3 4
## X-squared = 11.017, df = 1, p-value = 0.0009027
## 
## 
##  Chi-squared Test for Trend in Proportions
## 
## data:  r[id == i] out of n[id == i] ,
##  using scores: 1 2 3 4 5
## X-squared = 20.18, df = 1, p-value = 7.048e-06
```

```
x <- matrix(c(30, 54, 59, 383), ncol = 2)
chisq.test(x)
```

```
## 
##  Pearson's Chi-squared test with Yates' continuity correction
## 
## data:  x
## X-squared = 23.552, df = 1, p-value = 1.216e-06
```

```
x <- matrix(c(40, 195, 34, 38), ncol = 2)
chisq.test(x)
```

```
## 
##  Pearson's Chi-squared test with Yates' continuity correction
## 
## data:  x
## X-squared = 25.852, df = 1, p-value = 3.685e-07
```

#### High-impact factor journals

```
sel<-c("Anesth Analg 2017","Int J Cardiol 2017","J Consult Clin Psychol 2015","Ann Emerg Med 2012",
        "Scand J GastroEnterol 2013","Age Ageing 2014", "J Dent Res 2015","Int J Surgery 2017","PLOS One 2015",
        "J Psychosom Res 2011","J Psychosom Res 2015", "Acta Psych Scand 2017","Ann Surg 2014","Trials 2013",
        "BMJ Open 2011","Br J Anesth 2016","JRSM Open 2014")
ds.if<-ds[ds$study %in% sel,]
dim(ds.if)
```

```
## [1] 17 15
```

```
rma.arcsine(ds.if$n.reg.trials,ds.if$n.trials)
```

```
##            pIV     pIV_L     pIV_U      pREM    pREM_L    pREM_U      p0BB
## [1,] 0.5171657 0.5003204 0.5339916 0.5680849 0.4432943 0.6886338 0.5680709
##         p0BB_L    p0BB_U       pBB     pBB_L     pBB_U p0BiasCor
## [1,] 0.4565688 0.6761857 0.5670676 0.4555601 0.6752377 0.5525065
##      p0LBiasCor p0UBiasCor  pBiasCor pLBiasCor pUBiasCor       rho
## [1,]  0.4409725  0.6614277 0.5525764 0.4410423 0.6614943 0.2139148
##           tau2        Q
## [1,] 0.2679613 822.1987
```

```
sum(ds.if$n.trials)
```

```
## [1] 3383
```

```
ds.notif<-ds[!(ds$study %in% sel),]
dim(ds.notif)
```

```
## [1] 23 15
```

```
rma.arcsine(ds.notif$n.reg.trials,ds.notif$n.trials)
```

```
##            pIV     pIV_L     pIV_U      pREM   pREM_L    pREM_U      p0BB
## [1,] 0.4541446 0.4408705 0.4674513 0.5081566 0.418698 0.5973526 0.5082718
##         p0BB_L    p0BB_U       pBB     pBB_L     pBB_U p0BiasCor
## [1,] 0.4253259 0.5909891 0.5034903 0.4206011 0.5862829 0.5047196
##      p0LBiasCor p0UBiasCor  pBiasCor pLBiasCor pUBiasCor       rho
## [1,]  0.4218151  0.5874936 0.5023012 0.4194273 0.5851115 0.1591239
##           tau2       Q
## [1,] 0.1851841 957.697
```

```
sum(ds.notif$n.trials)
```

```
## [1] 5390
```
