# Supplementary material for: Registration of published randomized trials: a systematic review and meta-analysis
Source: BMC Med. 2018 Oct 16;16:173. doi: 10.1186/s12916-018-1168-6 (PMC6190546; doi:10.1186/s12916-018-1168-6)
Supplement: Supplementary file 3 — PRISMA checklist. (DOCX 149 kb) [file 12916_2018_1168_MOESM3_ESM.docx]

# Additional file 1

## Table S1: Reasons for exclusion after full-text screening of article

| Study | Reason |
| --- | --- |
| 1. Hannink. Ann Surg. 2013 | cannot determine how many trials were not registered |
| 1. Howard. PLOS One. 2017 | cannot determine how many trials were not registered |
| 1. Wager. BMJ. 2013 | data on journal policy but not on RCTs |
| 1. Korevaar. BMJ Open. 2014 | diagnostic accuracy studies |
| 1. Nijjar. BJOG. 2017 | Method to assess registration unclear |
| 1. Agha. Int J Surg. 2007 | middle of the range of years covered was prior to 2005 |
| 1. Kaplan. PLOS One. 2015 | middle of the range of years covered was prior to 2005 |
| 1. Khan. Arthritis Rheumatol. 2012 | middle of the range of years covered was prior to 2005 |
| 1. Vera-Badillo. Ann Oncol. 2013 | middle of the range of years covered was prior to 2005 |
| 1. Babu. Phys Ther. 2014 | not limited to RCTs* |
| 1. Dal-Re. J Clin Epidemiol. 2016 | not limited to RCTs* |
| 1. Huser. JAMIA. 2013 | not limited to RCTs* |
| 1. van den Bogert. PLOS One. 2016 | not limited to RCTs* |
| 1. Wieseler. BMJ. 2011 | primary studies from health technology assessments prepared by IQWiG (not all RCTs were published) |
| 1. Ewart. Ann Fam Med. 2009 | reporting of registration number only |
| 1. Falk Delgado. Sci Rep. 2017 | reporting of registration number only |
| 1. Fleming. PLOS One. 2015 | reporting of registration number only |
| 1. Freshwater. J Plast Reconstr Aesthet Surg. 2013 | reporting of registration number only |
| 1. Harriman. Trials. 2016 | reporting of registration number only |
| 1. Hopewell. Trials. 2013 | reporting of registration number only |
| 1. Huic. PLos One. 2011 | reporting of registration number only |
| 1. Jull. Int J Nursing Studies. 2015 | reporting of registration number only |
| 1. Lee. J Trauma Acute Care Surg. 2013 | reporting of registration number only |
| 1. Li. PLos One. 2012 | reporting of registration number only |
| 1. Lu. Resp Care. 2013 | reporting of registration number only |
| 1. Myles. Br J Anesth. 2013 | reporting of registration number only |
| 1. Rayhill. Neurology. 2015 | reporting of registration number only |
| 1. Reveiz. Cad Saude Publica. 2013 | reporting of registration number only |
| 1. Reveiz. J Clin Epidemiol. 2010 | reporting of registration number only |
| 1. Rongen. J Bone Joint Surg. 2016 | reporting of registration number only |
| 1. Rosenthal. Ann Surg. 2013 | reporting of registration number only |
| 1. Perez-Mana. Med Clin. 2012 | review |
| 1. Su. Trials. 2015 | sample of registered acupuncture RCTs |
| 1. Lee. Rev Panam Salud Publica. 2016 | sample of registered cancer clinical trials |
| 1. Huser. PLOS One. 2013 | sample of registered clinical trials |
| 1. Phillips. JAMA Intern Med. 2016 | studies supporting FDA approval of novel high-risk cardiovascular devices (not all RCTs were published) |
| 1. Van Lent. J Clin Epidemiol. 2015 | submitted (not published) RCT reports |
| 1. Reveiz. Trials. 2007 | survey regarding trialists’ attitude towards registration |
| 1. Wieseler. PLOS Medicine. 2013 | trials for which the IQWiG had requested and received full Clinical Study Reports from drug manufacturers (not all RCTs were published) |
| 1. Smith. Pain. 2013 | trials indexed in the RReACT database, and by design all are registered on ClinicalTrials.gov |
| 1. Tompson. BMJ Open. 2016 | trials supported by the NIHR Oxford Biomedical Research Centre and Musculoskeletal Biomedical Research Unit (not all RCTs were published and not limited to RCTs) |
| 1. Lassman. BMJ Open. 2017 | trials supporting FDA drug approvals (not all RCTs were published) |
| 1. Miller. BMJ Open. 2016 | trials supporting FDA drug approvals (not all RCTs were published) |
| 1. Phillips. Trials. 2017 | trials supporting FDA drug approvals (not all RCTs were published) |
| 1. Alturki. J Clin Epidemiol. 2017 | trials that provided a registration number in the full text |
| 1. Rasmussen. Trials. 2009 | unit of analysis is the RCT report not the RCT |

* None of the 4 studies not limited to RCTs reported relevant data on registration for the subgroup of RCTs.

## Table S2: Prevalence of registration among 12 studies that examined RCTs published in a single year

| Study | RCT publication year | Number  of trials | Number of  registered trials | Proportion of  registered trials (95%CI) | Number of  prospectively registered trials | Proportion of  prospectively registered trials (95%CI) |
| --- | --- | --- | --- | --- | --- | --- |
| Hamm 2010 | 2007 | 300 | 69 | 0.23 (0.18; 0.28) | NA |  |
| Kunath 2011 | 2009 | 106 | 63 | 0.59 (0.49; 0.69) | NA |  |
| Dekkers 2011 | 2009 | 133 | 88 | 0.66 (0.57; 0.74) | 9 | 0.07 (0.03; 0.12) |
| Pinto 2013 | 2009 | 200 | 67 | 0.34 (0.27; 0.4) | 12 | 0.06 (0.03; 0.1) |
| Reveiz 2012 | 2010 | 526 | 89 | 0.17 (0.14; 0.2) | 21 | 0.04 (0.02; 0.06) |
| van de Wetering 2012 | 2010 | 302 | 185 | 0.61 (0.56; 0.67) | NA |  |
| Gates 2018 | 2012 | 300 | 139 | 0.46 (0.41; 0.52) | NA |  |
| Emdin 2015 | 2012 | 191 | 92 | 0.48 (0.41; 0.55) | NA |  |
| Bonnot 2016 | 2013 | 183 | 113 | 0.62 (0.54; 0.69) | 53 | 0.29 (0.23; 0.36) |
| Cybulski 2016 | 2013 | 101 | 41 | 0.41 (0.31; 0.51) | 13 | 0.13 (0.07; 0.21) |
| Smail-Faugeron 2015 | 2013 | 317 | 73 | 0.23 (0.19; 0.28) | 13 | 0.04 (0.02; 0.07) |
| Rosati 2016 | 2013 | 20 | 20 | 0.98 (0.83; 1) | 18 | 0.9 (0.68; 0.99) |

## Figure S1: Funnel plot for the log odds of trial registration


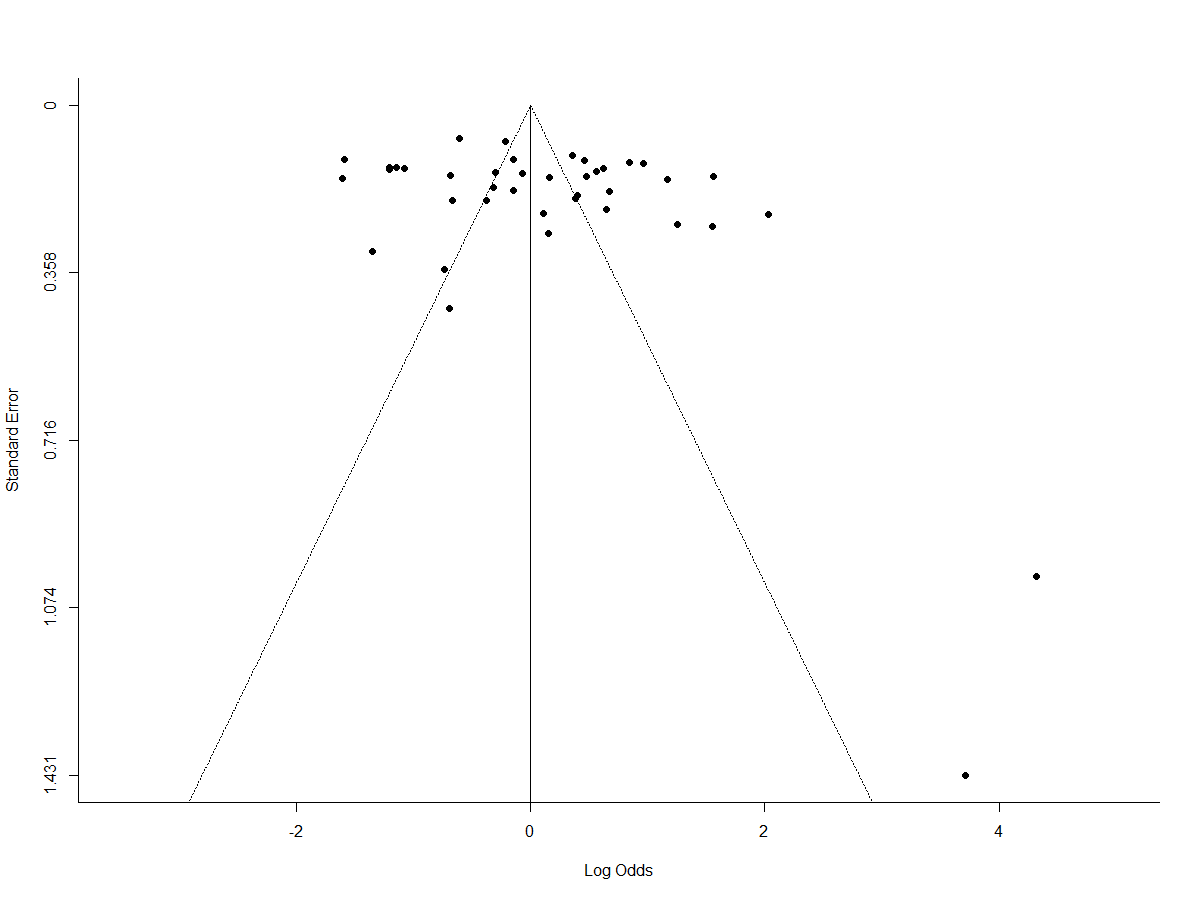


Funnel plots are typically used with treatment effect estimates from comparative studies. It is suspected that studies are not reported on the basis of their statistical significance, giving rise to an asymmetric funnel plot. In the context of our meta-analysis, if studies with smaller proportions of registered trials are less likely to report this outcome, then we can also expect an asymmetric funnel plot for the log odds of registration, with the left-hand side of the funnel omitted. In fact, smaller studies are more likely to have such extreme results because of sampling error and are thus not reported.

## Figure S2: Proportion of registered RCTs among published RCTs that started enrolment after 2005


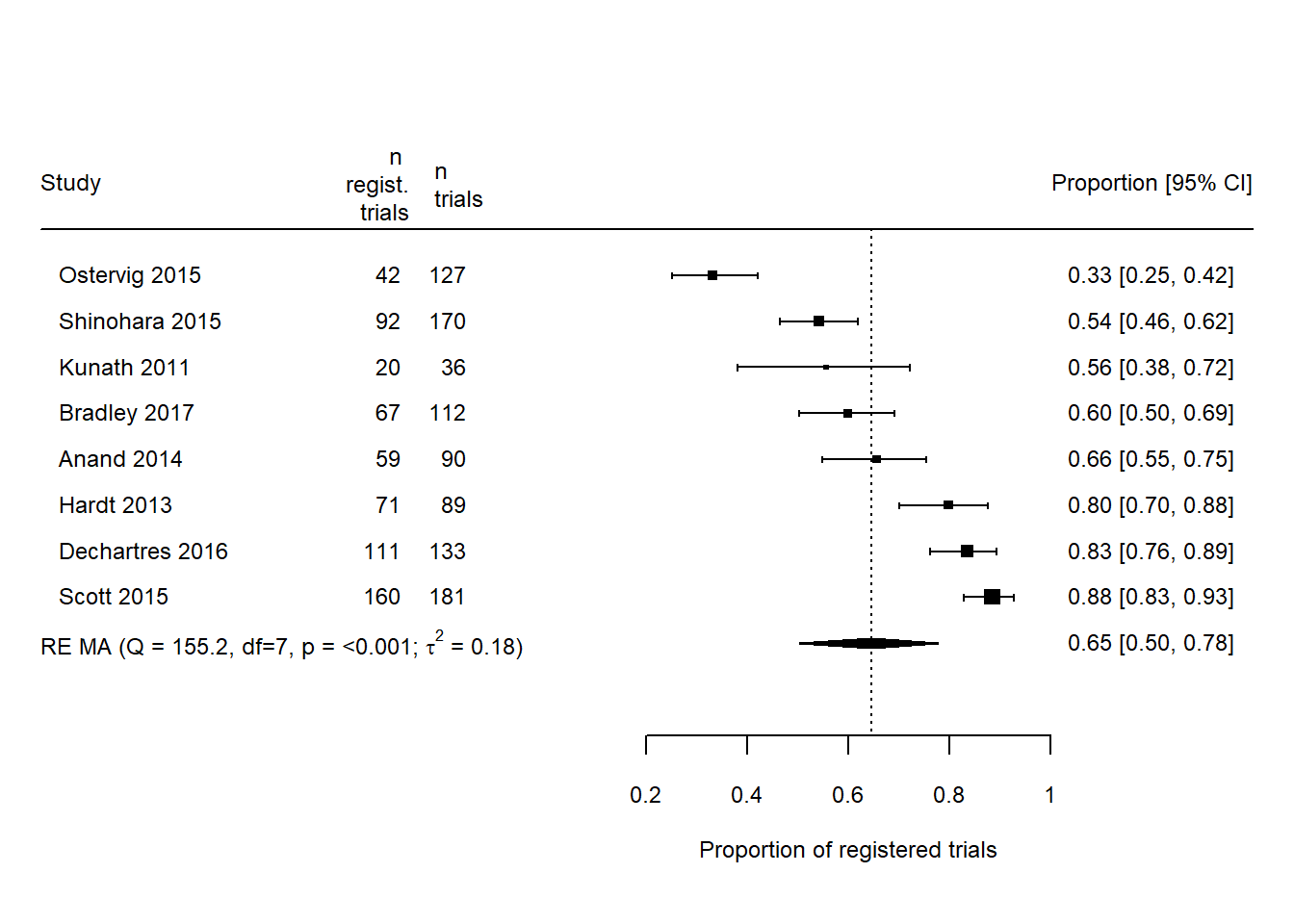


*Figure S3: Meta-regression analysis of prevalence of prospective trial registration in relation to publication year.
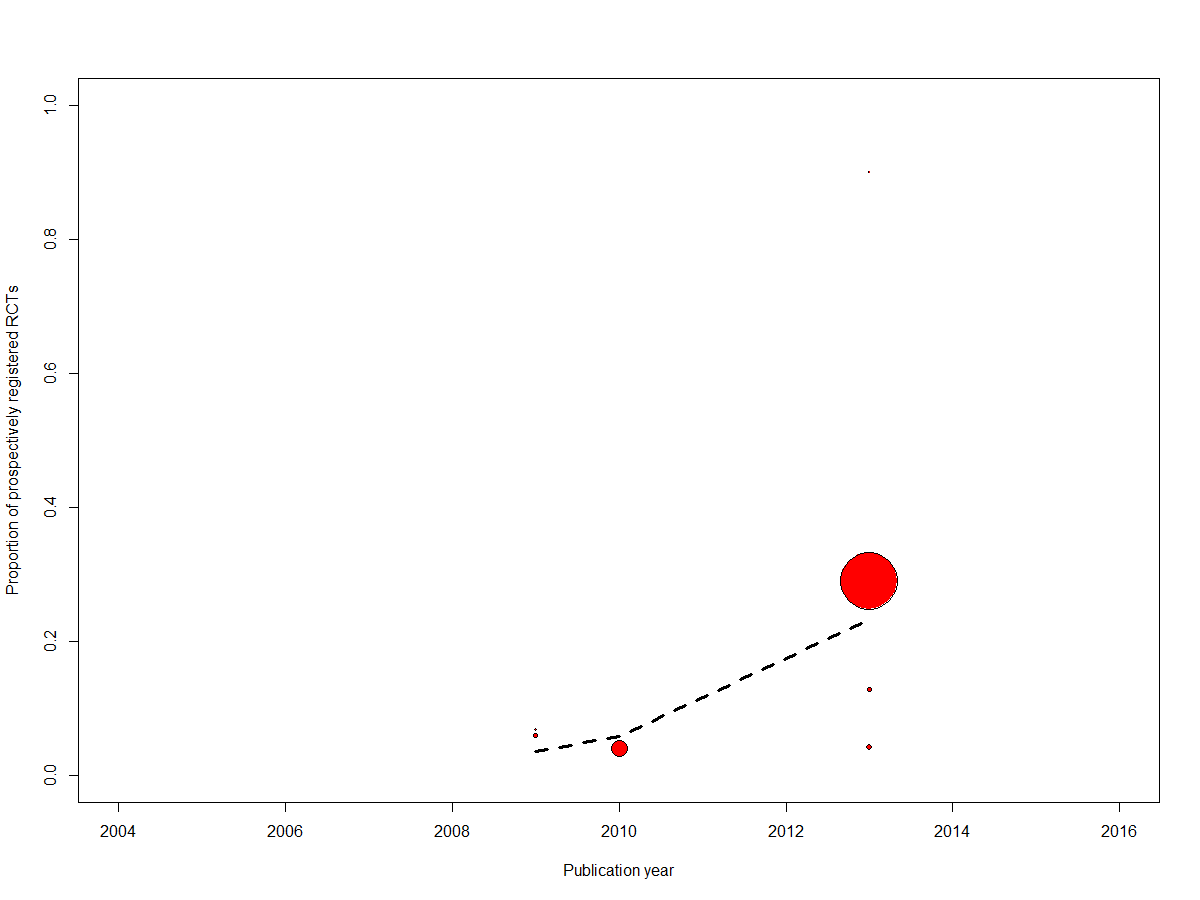
*

The analysis is based on 7 studies that examined RCTs published in a single year. Each circle represents one study and the size of the circle represents the weight given to the study in meta-regression. The black dashed line corresponds to a meta-regression model across these 7 studies. While our numbers are small, the analysis suggests that the proportion of prospectively registered trials increased over time, from 3% in 2009 to 21% in 2013 (18% increase, p=0.04).
